# Effects of Five Daily Activities on Harmonic Analysis of the Radial Pulse

**DOI:** 10.1155/2020/6095674

**Published:** 2020-09-27

**Authors:** Pan-Jen Chen, Han-Kuei Wu, Po-Chi Hsu, Lun-Chien Lo, Hen-Hong Chang

**Affiliations:** ^1^Department of Chinese Medicine, China Medical University, Taichung 40402, Taiwan; ^2^School of Post-Baccalaureate Chinese Medicine-Internal Medicine, China Medical University, Taichung 40402, Taiwan; ^3^Department of Traditional Chinese Medicine, Kuang Tien General Hospital, Taichung, Taiwan; ^4^Department of Chinese Medicine, China Medical University Hospital, Taichung 40447, Taiwan; ^5^Graduate Institute of Integrated Medicine, China Medical University, Taichung 40402, Taiwan; ^6^Chinese Medicine Research Center, China Medical University, Taichung 40402, Taiwan

## Abstract

All daily physiological activities have some effects on the body, and traditional Chinese medicine believes that pulse diagnosis can reflect the circulation of qi and blood throughout the body. This study aimed to explore the effects of five physiological activities, namely, sleep, exercise, ingestion, defecation, and shower, on pulse waves of the radial artery. Thirty test subjects were recruited for the study, and a wearable pulse signal measurement device was used for self-measurement of radial artery pulses before and after various physiological activities. All collected data were subjected to fast Fourier analysis, which transformed each wave from its time domain to frequency domain of 10 harmonics to describe the changes in pulse waves. The results were as follows: exercise and sleep had larger but opposite effects on the pulse waves; defecation and sleep relaxed the body and had the same trend of effect on the pulse waves. Both exercise and ingestion require energy to proceed, and both exert a burden on the body, and the pulse waves showed the same trend of changes. In contrast, shower had a little effect on the pulse waves. Preliminary observation in this study showed that relaxation of the body could increase high-level harmonics, whereas stress could increase low-level harmonics. Further studies are warranted to unravel the physiological significance of this finding.

## 1. Background and Objectives

Pulse waves are a major basis of diagnosis in traditional Chinese medicine (TCM), through which the conditions of the visceral organs and meridians of the body can be understood. Pulse waves of the radial artery can reflect the circulation of the qi and blood throughout the body.

All daily physiological activities can affect the body and induce changes of energy distribution within visceral organs and meridians and, in turn, changes in pulse waves. Thus, the effects of daily physiological activities on the body can be assessed by measuring and analyzing the pulse waves, through which theories of TCM can be applied. The use of modern technology and data analysis to understand the wisdom of TCM can help it move towards evidence- and data-based medicine. We used five daily physiological activities, namely, sleep, exercise, ingestion, defecation, and shower, to study their effects on pulse diagnosis.

Sleep is the most important way of rest for the human body. Its objectives are fatigue relieving, spirit recovering, and mind–body repairing. Among the previously mentioned five activities, sleep is the most static activity that allows the defensive qi back to yin for rest and restoration, and exercise is the most active activity, which can increase cardiac output and activate the meridians. The skeletal muscles require a large amount of blood supply during exercise, so more blood is diverted to the skeletal muscles and less is sent to the visceral organs.

Ingestion is an important activity for maintaining life and is the most important way to acquire materials for the body. Defecation, the activity of eliminating wastes from the large intestine, is the most important way to get rid of wastes from the body.

Taking a warm bath can relieve fatigue of the body and recover one's spirit. In terms of TCM, it can help the circulation of the qi and blood. However, the possible effects of taking a warm bath on the pulse waves and the possible changes in the pulse waves that make one feel relieved remain to be explored.

Because the effects of physiological activities on pulse diagnosis have not been clarified, the present study that used five common physiological activities to explore the relationship between these activities and changes in pulse diagnosis can further the integration between pulse diagnosis theory in TCM and modern physiology and offer more scientific explanation on pulse diagnosis.

## 2. Methods

### 2.1. Experimental Design and Protocol

Thirty healthy young adults aged 20–25 years were recruited, and their pulse waves were measured before and after performing five different physiological activities to explore the effects of these activities on pulse waves. This study was approved by the Institutional Review Board of China Medical University and Hospital Research Ethics Committee (approval no.: CMUH107-REC2-144). Written consent for participation was obtained from the test subjects.

### 2.2. Experimental Equipment and Evaluation Method

**Figure 1:**
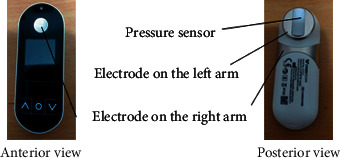
Illustration of the Freescan.

**Figure 2:**
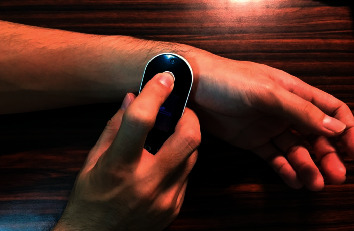
Freescan measurement: the right hand is used to hold the Freescan instrument to measure pulse site on the left hand.

The measuring instrument used was Freescan (Maisense Inc., product no. 311020; Zhubei City, Hsinchu County, Taiwan) (Figure 1), which can simultaneously measure pressure waves of the left radial artery and can be used as Lead I of electrocardiography (ECG-I). For measuring, the right hand is used to hold the Freescan instrument and press it on the radial side of the left hand to let the piezoelectric material record pressure changes in the artery (Figure 2). This pressure change would vary in accordance with the flow speed of the arterial blood, so the pressure waves can reflect the changes of pulse waves. Different electric pressures of right and left hands can be measured via electrodes of the instrument to obtain ECG-I. Heart rate cycle of each heart beat can be calculated with ECG-I. This method was used to dissect each pulse wave, which was then used by fast Fourier analysis to obtain different characteristics of each pulse wave.

### 2.3. Experimental Protocol

Measurements before and after taking a shower: before taking a shower, subjects sat still for 3 min and took one measurement of the pulse waves in sitting position and then took a shower for 15–20 min. After the shower, the subjects dried themselves and got dressed to keep the body warm. If the hair was washed, the subjects wrapped their hair with a towel, sat still for 3 min, and took another measurement of pulse waves.

Measurements before and after defecation: Because the need for defecation may be urgent, there was no need to sit still for 3 min before taking the measurement of pulse waves. One measurement was taken while sitting on the toilet before defecation actually happened. Another measurement was taken right after defecation was done while the subject was still sitting on the toilet. This ensured that pulse wave measurement was taken most recent to before and after the physiological activity.

Measurements before and after ingestion: before taking a meal, the subjects sat still for 3 min and took one measurement of the pulse waves in sitting position. All subjects ate the same type of boxed lunch. After the meal, the subjects sat still for 3 min and took another measurement of pulse waves in sitting position.

Measurements before and after sleep: before sleep, the subjects sat still on a bed for 3 min and took one measurement of pulse waves. After waking up the next morning, the subjects sat still on the bed for 3 min and took another measurement.

Measurement before and after exercise: before exercise, the subjects sat still for 3 min and took one measurement of pulse waves. To simplify the exercise, all subjects jogged on a treadmill at the speed of 8 km/h for 10 min. After the exercise and before the heart rate returned to normal, the subjects took another measurement of pulse waves.

### 2.4. Pulse Wave Analysis

Single pulse waves were cut out using a computer program from measured data of ECG-I and pulse wave data obtained by the instrument and subjected to fast Fourier transform using Matlab [1], which transforms each pulse wave from its time domain to frequency domain of 10 harmonic amplitudes [2] to describe the changes in pulse waves.

The heart pumps blood by contraction, and the blood circulates throughout the body via blood vessels [3, 4]. From anatomical structures, the blood pumped from the heart takes a big turn at the aortic arch. The elastic arteries can store the kinetic energy of the blood in the form of elastic potential energy [5–8]. During the diastolic phase of the heart, the elastic potential energy of the arteries is released and the blood rebounds to close the aortic valve. Because the arteries distribute the blood throughout the body, all conditions of the body can affect the arteries. These effects in the radial artery are expressed through pulse waves.

The Freescan is a portable instrument that requires one hand to push on the other hand for measurement. The pressure applied varies according to different people and different times. There is no way to obtain the exact pressure applied or the exact pulse pressure mean. Moreover, the harmonic energy measured before and after a physiological activity cannot be compared directly. To explore the difference of harmonic energy measured before and after a physiological activity, this study used the total energy of all harmonics as the base to differentiate the harmonic weighting (HWt). The amplitudes of all frequencies are added to obtain the total energy, and then, the ratio of each harmonic to the total energy is calculated.

(1) $$\begin{array}{c} \text{HWt}=\frac{\text{Harmonic energy}}{\text{Total energy of all harmonic}}. \end{array}$$

### 2.5. Statistical Analysis

Pulse waves of each individual is different; even in the same individual, pulse waves are not the same every time. To eliminate individual differences, this study used multiple pulse waves from 30 individuals to explore the effects of physiological activities on pulse waves. However, the difficulty of this study was that no statistical method completely fitted this pulse wave study. As the harmonics of pulse wave are not normally distributed, this study used Wilcoxon sign rank test which is a kind of nonparametric statistics to determine if a physiological intervention has an effect on the pulse wave.

## 3. Results

Single pulse wave was cut out using a computer program from ECG-I and pulse wave data obtained by the instrument and analyzed using fast Fourier transform to obtain different harmonics. In some subjects, the pulse waves were too weak or the ECG signals were not strong enough for their pulse waves to be detected by the instrument. Thus, different subjects could have different number of pulse waves measured before and after a physiological signal, which could result in some invalid pulse waves. To compare the effect of a physiological activity on pulse waves, we should choose the most recent continuous 15 pulse waves before and after the activity to eliminate the interferences of time. This method not only takes into consideration the variability within the subject but also increases the sample numbers. Since each subject has the same amount of data, it may eliminate the interferences caused by individual subject who was recorded more successful pulse waves. Unfortunately, due to the sensitivity of Freescan instrument, the signal strength of pulse waves, and operational issues of participant, not all subjects had received 15 pulse waves before and after a physiological activity, so the missing data of the subjects were excluded from our analysis. Therefore, in this study, we only analyzed 19 cases in shower, defecation, and ingestion, 17 cases in sleep, and 20 cases in exercise. Although there may be no data in certain fields in some subjects, the statistical analysis was not affected.

To understand the relative proportion of harmonics in the overall condition of all subjects, this study collected pulse waves measured before all physiological activities, some after sitting still for 3 min, for calculation of the baseline of each HWt. The results are shown in Table 1.

Table 2 lists the differences of all HWts (dHWt) before and after five physiological activities. This is the average of dHWts from all subjects.

(2) $$\begin{array}{c} \text{dHWt}=\text{HWt after activity}-\text{HWt before activity.} \end{array}$$

Nineteen subjects performed the shower activity, and 15 pulse waves were measured before and after the activity; thus, there were 285 sets of data. Furthermore, 19 subjects performed the defecation and ingestion activities, and each had 285 sets of data. In addition, 17 subjects performed the sleep activity and 20 performed the exercise activity, with 255 and 300 sets of data, respectively. Because the percentage of different harmonics over total pulse wave energy were different, to quantify the effects of different physiological activities on different harmonics, we used the baseline HWt of each subject before activity as the standard to calculate standardization of dHWt and the results (sHWt) are shown in Table 3.

(3) $$\begin{array}{c} \text{sHWt}=\frac{\text{dHWt}}{\text{HWt before activity}}. \end{array}$$

The effects of five different physiological activities on harmonics are shown in Table 4.

## 4. Discussion

Pulse waves vary among different subjects and even within the same subject owing to different times of measurement. Although the subjects were asked to sit still for 3 min before taking measurements, many daily physiological activities may have long-lasting effects on pulse waves that may not recover after sitting still for 3 min.

Analysis of harmonics is a tool that can rapidly quantify various pulse waves. This study used 1^st^–10^th^ harmonics to describe one pulse wave that can be rapidly used to compare and quantify changes of pulse waves before and after physiological activities. Table 1 shows the HWt of preactivity pulse waves; in general, the lower the harmonic, the higher the HWt, and the lower the HWt, the lower the coefficient of variance. This study defined low-level harmonics as those with HWt CV < 0.4 (1^st^–4^th^ harmonics), and the rest (5^th^–10^th^ harmonics) were high-level harmonics. The low-level harmonics are more difficult to be affected by different subjects and different times.

All five physiological activities, namely, shower, defecation, ingestion, sleep, and exercise, have an effect on pulse waves. Although shower affected 6 HWts, none of them exceeded 10%, which is quite a small change in pulse waves. This may be the reason why shower has not been specifically mentioned in TCM literature previously.

Tables 3 and 4 show that defecation affected 6 HWts, and the most affected 7^th^ and 8^th^ HWts increased by both 14.8%. The percentages for higher-levels harmonics over the total energy increased, whereas the 2^nd^ HWt decreased by 3.8%. Ingestion affected 9 HWts, and the 2^nd^ HWt increased by 10.1%; this is a great effect on low-level harmonics. The 5^th^–10^th^ HWts decreased by at least 20%. Ingestion caused an obvious decrease in the energy of high-level harmonics but increased that of the 2^nd^ harmonic. It can be observed from the harmonic analysis that the two gastrointestinal activities, defecation and ingestion, have opposite effects on pulse waves: defecation decreased low-level harmonics and increased high-level harmonics, whereas ingestion increased low-level harmonics and decreased high-level harmonics.

Tables 3 and 4 show that sleep affected nine harmonics; the HWts of the most affected 8^th^, 9^th^, and 10^th^ increased by 22.2%, 19.6%, and 14.8%, respectively. The percentage for higher-level harmonics over total energy increased, whereas the 1^st^ HWt decreased by 3.4% and the 2^nd^ HWt decreased by 5.9%. Exercise remarkably affected nine HWts; the 1^st^ and the 2^nd^ HWt increased by 20.9% and 27.6%, respectively, and the 3^rd^–8^th^ HWts were greatly increased. Sleep is the most static state of the human body, and exercise is the most active state; sleep and exercise had opposite effects on pulse waves. Static state decreased low-level harmonics but increased high-level ones, whereas active state increased low-level harmonics but decreased high-level ones.

## 5. Conclusion

Harmonic analysis revealed that defecation and sleep have the same trend of effects on pulse waves, with greater effect exerted by sleep. Exercise and ingestion also have the same trend of effects on pulse waves, with greater effect exerted by exercise. It is postulated that the body experiences tension and relaxation before and after defecation, respectively, which is similar to a good night's sleep after a hard day of work. However, exercise and ingestion both exert burden on the body and the body needs to use energy to deal with the external stress or ingested food; thus, the pulse waves may have the same trend of responses. The trends observed in this study are as follows: relaxation of the body may increase high-level harmonics, and stress may increase low-level ones. The physiological significance of this finding warrants further studies.

## Figures and Tables

**Table 1:** Baseline of harmonic weighting (HWt).

*N* = 2349	1^st^ HWt	2^nd^ HWt	3^rd^ HWt	4^th^ HWt	5^th^ HWt	6^th^ HWt	7^th^ HWt	8^th^ HWt	9^th^ HWt	10^th^ HWt
Mean	44.45%	26.95%	12.84%	7.91%	4.01%	1.81%	0.89%	0.52%	0.35%	0.27%
SD	10.72%	5.99%	3.60%	2.94%	2.30%	1.46%	0.89%	0.65%	0.52%	0.46%
CV	0.24	0.22	0.28	0.37	0.57	0.80	1.01	1.24	1.49	1.72

**Table 2:** Differences of each HWt before and after various activities.

Activity (%)	dHWt									
	1^st^	2^nd^	3^rd^	4^th^	5^th^	6^th^	7^th^	8^th^	9^th^	10^th^
Shower (*n* = 19)	1.30^*∗∗*^	0.63	−0.66^*∗∗*^	−0.32^*∗∗*^	−0.42^*∗∗*^	−0.23^*∗∗*^	−0.10^*∗*^	−0.02	−0.03	−0.01
Defecation (*n* = 19)	−0.53	−0.98^*∗*^	−0.01	0.67^*∗∗*^	0.38^*∗∗*^	0.22^*∗∗*^	0.12^*∗∗*^	0.06^*∗∗*^	0.02	0.001
Ingestion (*n* = 19)	1.10^*∗*^	2.53^*∗∗*^	−0.71^*∗∗*^	−0.31	−0.90^*∗∗*^	−0.58^*∗∗*^	−0.39^*∗∗*^	−0.13^*∗∗*^	−0.09^*∗∗*^	−0.07^*∗∗*^
Sleep (*n* = 17)	−1.50^*∗∗*^	−1.34^*∗∗*^	0.28	0.41^*∗*^	0.47^*∗∗*^	0.31^*∗∗*^	0.20^*∗∗*^	0.15^*∗∗*^	0.07^*∗∗*^	0.06^*∗∗*^
Exercise (*n* = 20)	8.87^*∗∗*^	6.79^*∗∗*^	−7.01^*∗∗*^	−3.67^*∗∗*^	−2.90^*∗∗*^	−1.51^*∗∗*^	−0.51^*∗∗*^	−0.12^*∗∗*^	−0.004^*∗*^	0.04

^*∗*^ *p* < 0.05, ^*∗∗*^*p* < 0.01.

**Table 3:** Differences of each sHWt before and after various physiological activities.

Activity	sHWt (%)									
	1^st^	2^nd^	3^rd^	4^th^	5^th^	6^th^	7^th^	8^th^	9^th^	10^th^
Shower (*n* = 19)	3^*∗∗*^	2.8	−4.4^*∗∗*^	−4.6^*∗∗*^	−8.8^*∗∗*^	−9.3^*∗∗*^	−8.5^*∗*^	−3.5	−8.4	−6.2
Defecation (*n* = 19)	−1.2	−3.8^*∗*^	−0.1	9.7^*∗∗*^	9.3^*∗∗*^	12.5^*∗∗*^	14.8^*∗∗*^	14.8^*∗∗*^	7.9	−0.5
Ingestion (*n* = 19)	2.6^*∗*^	10.1^*∗∗*^	−4.9^*∗∗*^	−4.3	−20.1^*∗∗*^	−26.8^*∗∗*^	−37.3^*∗∗*^	−29.3^*∗∗*^	−31.8^*∗∗*^	−33.2^*∗∗*^
Sleep (*n* = 17)	−3.4^*∗∗*^	−5.9^*∗∗*^	2	5.7^*∗*^	9.9^*∗∗*^	11.4^*∗∗*^	14.6^*∗∗*^	22.2^*∗∗*^	19.6^*∗∗*^	29.5^*∗∗*^
Exercise (*n* = 20)	20.9^*∗∗*^	27.6^*∗∗*^	−43.9^*∗∗*^	−51.8^*∗∗*^	−71.3^*∗∗*^	−76.3^*∗∗*^	−63^*∗∗*^	−32.1^*∗∗*^	−2^*∗*^	22.8

^*∗*^ *p* < 0.05, ^*∗∗*^*p* < 0.01.

**Table 4:** Effects of five different physiological activities on harmonics.

Effect		
Activity	Energy increased	Energy decreased
Shower (*n* = 19)	1^st^ harmonic	3^rd^, 4^th^, 5^th^, 6^th^, and 7^th^ harmonic
Defecation (*n* = 19)	4^th^, 5^th^, 6^th^, 7^th^, and 8^th^ harmonic	2^nd^ harmonic
Ingestion (*n* = 19)	1^st^ and 2^nd^ harmonic	3^rd^, 5^th^, 6^th^, 7^th^, 8^th^, 9^th^, and 10^th^ harmonic
Sleep (*n* = 17)	4^th^, 5^th^, 6^th^, 7^th^, 8^th^, 9^th^, and 10^th^ harmonic	1^st^ and 2^nd^ harmonic
Exercise (*n* = 20)	1^st^ and 2^nd^ harmonic	3^rd^, 4^th^, 5^th^, 6^th^, 7^th^, 8^th^, and 9^th^ harmonic

## Data Availability

Data used to support the study are included within the article. No additional data are available.
